# Resveratrol targeting MDM2/P53/PUMA axis to inhibit colonocyte apoptosis in DSS-induced ulcerative colitis mice

**DOI:** 10.3389/fphar.2025.1572906

**Published:** 2025-04-30

**Authors:** Rui Tang, Ling Jiang, Quan Ji, Pengyuan Kang, Yuan Liu, Pengyu Miao, Xiaofan Xu, Mingxi Tang

**Affiliations:** 1 Department of Pathology, Yaan People’s Hospital, Yaan, China; 2 School of Basic Medical Sciences, Southwest Medical University, Luzhou, China; 3 Department of Anesthesiology Management, the Affiliated Hospital of Southwest Medical University, Luzhou, China; 4 Department of Microbiology and Infectious Disease Center, School of Basic Medical Sciences, Peking University, Beijing, China; 5 Department of Health Technology and Informatics, The Hong Kong Polytechnic University, Kowloon, China; 6 Department of Pathology, The Affiliated Hospital, Southwest Medical University, Luzhou, China; 7 Precision Medicine Center, Yaan People’s Hospital, Yaan, China

**Keywords:** ulcerative colitis, resveratrol, apoptosis, colonocyte, MDM2/P53/PUMA axis

## Abstract

**Background:**

Resveratrol, a naturally occurring polyphenolic compound found in grapes, berries, and traditional medicinal plants like Polygonum cuspidatum, has been used for centuries in traditional medicine systems for its anti-inflammatory, antioxidant, and cardioprotective properties. Ulcerative colitis (UC), a chronic inflammatory bowel disease, is characterized by intestinal barrier disruption due to excessive colonocyte apoptosis, leading to increased permeability and inflammation. Targeting apoptosis is a critical therapeutic strategy for UC.

**Aim of the study:**

This study aims to investigate the therapeutic potential of Resveratrol in ulcerative colitis (UC) by targeting excessive colonocyte apoptosis and intestinal barrier dysfunction. Specifically, we seek to elucidate the mechanisms through which Resveratrol modulates apoptosis-related pathways and evaluate its efficacy in restoring intestinal homeostasis and mitigating UC progression in both *in vivo* and *in vitro* models.

**Materials and Methods:**

We used dextran sulfate sodium (DSS) to induce UC in a mouse model. Colonic damage was assessed through colonic length measurement, histological examination, and immunofluorescence staining. Single-cell sequencing was employed to explore changes in the colonic immune microenvironment and cellular signaling pathways after Resveratrol treatment. *In vitro*, colonocytes isolated from healthy mouse colonic tissue were exposed to TGF-β to induce apoptosis. DNA fragmentation, mitochondrial membrane potential, and annexin V/propidium iodide staining were used to assess apoptosis. Additionally, we employed an Adeno-Associated Virus system to overexpress MDM2 in the colon and evaluate its protective role in DSS-induced UC.

**Results:**

Resveratrol treatment effectively repaired colonic damage in the UC mouse model by significantly increasing colon length, reducing inflammatory cell infiltration, and mitigating mucosal injury. Single-cell sequencing revealed that Resveratrol primarily targeted colonocytes, decreasing genes related to apoptosis and the P53 pathway. *In vitro*, Resveratrol reduced DNA fragmentation, apoptotic cell populations, and increased mitochondrial membrane potential in a dose-dependent manner. Furthermore, Resveratrol increased MDM2 expression, inhibiting P53 and downstream pro-apoptotic signaling. Nutlin-3a, an MDM2 inhibitor, reversed the anti-apoptotic effects of Resveratrol. Overexpression of MDM2 in the colon protected against DSS-induced damage.

**Conclusion:**

Resveratrol is an effective treatment for DSS-induced UC, primarily by inhibiting excessive apoptosis in colonocytes through the MDM2/P53/PUMA axis. MDM2 presents a promising therapeutic target for UC treatment.

## Introduction

Ulcerative colitis (UC) is a chronic non-specific inflammatory disease of the colon and rectum whose etiology is not very clear (Feuerstein et al., 2019; da Silva et al., 2014). The UC patients experience symptoms such as diarrhea, abdominal pain, rectal bleeding, and an urgent need to defecate (Conrad et al., 2014). The pathogenesis of UC is complex and involves a combination of genetic predisposition, environmental factors, and immune system dysregulation (Du and Ha, 2020; Lee et al., 2018).

A critical aspect of UC is the disruption of the intestinal barrier, which plays a vital role in maintaining gut homeostasis and protecting the body from harmful pathogens and toxins (Foerster et al., 2022). The intestinal barrier is composed of a single layer of epithelial cells, known as colonocytes, which are tightly joined together by protein complexes called tight junctions (Wu et al., 2021). These tight junctions regulate the permeability of the intestinal lining, ensuring that only essential nutrients and water pass through while keeping harmful substances out (Camilleri et al., 2012; Peterson and Artis, 2014).

In UC, the integrity of the intestinal barrier is compromised due to increased colonocyte apoptosis. Apoptosis is a natural process that helps maintain cellular homeostasis by eliminating damaged or unnecessary cells (Elmore, 2007). However, in the context of UC, excessive apoptosis of colonocytes leads to a weakened barrier, increased intestinal permeability, and subsequent translocation of bacteria and antigens into the underlying tissue (Wan et al., 2022). This breach in the barrier triggers an exaggerated immune response, resulting in chronic inflammation and further damage to the intestinal lining (Wan et al., 2022). The interplay between ulcerative colitis, intestinal barrier dysfunction, and colonocyte apoptosis is a key area of research, as understanding these mechanisms can provide insights into potential therapeutic targets.

Resveratrol, a natural polyphenolic compound found in grapes, red wine, and several other plants, has garnered significant attention for its potent anti-inflammatory, antioxidant, and anti-apoptotic effects (Shaito et al., 2020; de Sá Coutinho et al., 2018; Huo et al., 2022; Yoo and Kim, 2015). As a dietary supplement, resveratrol has been studied extensively for its potential therapeutic benefits in various chronic diseases, including cardiovascular diseases, cancer, and metabolic disorders (Galiniak et al., 2019). Recent research has also explored its potential role in the management of inflammatory bowel diseases, particularly ulcerative colitis (Shi et al., 2017).

Resveratrol exerts its beneficial effects through multiple mechanisms, including the inhibition of pro-inflammatory signaling pathways such as nuclear factor kappa B (NF-κB) and the activation of antioxidant defenses (Bagul et al., 2015; Bhattarai et al., 2016). Additionally, resveratrol can modulate apoptosis by regulating the expression of apoptotic proteins, thereby promoting the survival of healthy colonocytes and maintaining the integrity of the intestinal barrier (Asadollahi et al., 2022).

Although its protective effect against Ulcerative colitis has been confirmed by many studies, the mechanism behind it still lacks a deep and comprehensive understanding.

## Methods and materials

### Animals

All procedures involving animals were performed in strict accordance with China’s national regulations on the use of experimental animals and were approved by the Southwest Medical University Animal Welfare Review Committee (Approval No. 20211123-036). Mice were housed in a controlled environment with a 12-h light/dark cycle, maintained at 22°C ± 2°C with 50%–60% humidity, and provided with *ad libitum* access to food and water. Animal welfare was monitored daily by trained personnel, and humane endpoints were strictly followed. These endpoints included, but were not limited to, a loss of >20% body weight, severe lethargy, inability to access food or water, or signs of prolonged distress. Mice were sacrificed by CO2 asphyxiation, followed by cervical dislocation to ensure death, and all efforts were made to minimize suffering. The CO2 flow rate was carefully controlled to avoid discomfort, and euthanasia was performed in a dedicated, quiet area to reduce stress.

Resveratrol treatment experiment: Eighteen C57BL/6 male mice (25 ± 2 g) were randomly divided into three groups (n = 6 per group): the DSS group received 5% (w/v) dextran sulfate sodium salt (DSS, Catalog #60316ES25, YEASEN Biology) in drinking water from day 1 to day 7 (Chassaing et al., 2014); the DSS+NS group received DSS as above, followed by daily intraperitoneal (i.p.) injections of normal saline (NS) from day 8 to day 24; and the DSS+Resveratrol group received DSS as above, followed by daily i.p. injections of 50 mg/kg Resveratrol (Catalog #R5010, Sigma) from day 8 to day 24. On day 8, mice in the DSS group were sacrificed, while the remaining groups (DSS+NS and DSS+Resveratrol) were treated until day 25, after which they were sacrificed. Colonic tissues were collected, washed in cold phosphate-buffered saline (PBS), and processed for downstream analysis.

MDM2 overexpression experiment: Plasmids pAAV-CAG-sec-GFP and pAAV-CAG-sec-MDM2 were constructed by inserting GFP and mouse MDM2 cDNAs into AAV serotype 2 vectors, and recombinant AAV (rAAV) particles were produced by co-transfecting HEK 293-T cells with the AAV plasmids and an enhancer plasmid using polyethylenimine (PEI) transfection reagent, followed by culture in DMEM supplemented with 10% fetal bovine serum (FBS) at 37°C with 5% CO2. Forty-eight hours post-transfection, cells were harvested, subjected to three freeze-thaw cycles, and centrifuged at 8,000 × g for 15 min, after which the supernatant was collected, purified using HiTrap heparin column chromatography (Sigma), dialyzed in PBS (pH 7.4) containing 1 mM MgSO4, and concentrated using a 100K-MicroSep centrifugal concentrator (Life Technologies). Viral titers were quantified by quantitative PCR (qPCR) using AAV-specific primers, adjusted to a final concentration of 1.5 × 10^12^ viral genomes/mL, and stored at −80°C until use (Ibreljic et al., 2024). For the animal experiment, twelve C57BL/6 male mice (25 ± 2 g) were randomly divided into two groups (n = 6 per group): the AAV-GFP group received 1 × 10^11^ viral genomes of AAV-GFP intravenously (i.v.), and the AAV-MDM2 group received 1 × 10^11^ viral genomes of AAV-MDM2 i.v. Four weeks post-injection, both groups received 5% (w/v) DSS in drinking water for 7 days, after which the mice were sacrificed on day 7 post-DSS induction.

### Single-cell sequencing

Colonic tissues from the DSS+NS and DSS+Resveratrol groups (n = 2 per group) were collected, washed in Hank’s Balanced Salt Solution (HBSS) containing 5 mM dithiothreitol (DTT) for 10 min at 25°C on a platform rocker to remove mucus, and further washed in DMEM medium supplemented with 10% FBS (Thermo Fisher), 150 U/mL penicillin-streptomycin (PS), 200 ng/mL amphotericin B (MCE), 15 μg/mL gentamicin sulfate (Sigma), and 1.5 mM HEPES (Sigma) for 5 min at 25°C on a platform rocker. Tissues were minced and digested in 500 µL of digestion buffer (DMEM containing 1 mg/mL collagenase IV, 0.1 mg/mL DNase I, and 20 μg/mL DNase I) at 30°C for 1 h on a shaking platform at 200 rpm, after which the cell suspension was filtered through a 75 μm cell strainer (Corning), washed with PBS, and resuspended in DMEM medium containing 0.1% bovine serum albumin (BSA) at a concentration of 1 × 10^6^ cells/mL. Single-cell suspensions were processed using the 10x Genomics Chromium Single Cell 3′ Reagent Kit (v3) according to the manufacturer’s instructions, and libraries were sequenced on an Illumina NovaSeq 6000 platform with a target depth of 50,000 reads per cell (Ziegenhain et al., 2017). For cell type identification, marker genes were selected based on established literature and validated using differential expression analysis (adjusted p-value <0.05, Benjamini-Hochberg correction). Pathway enrichment analysis was performed using the Gene Ontology (GO) and Kyoto Encyclopedia of Genes and Genomes (KEGG) databases, with significance thresholds set at p < 0.05 and false discovery rate (FDR) <0.1.

### Colonocyte isolation

Healthy C57B/L mice were sacrificed by CO2 asphyxiation, and the proximal colon was isolated and cleaned in PBS. The tissue was cut into 3 mm fragments and washed with pre-cold PBS repeated 5 times. The fragments were then incubated in PBS solution (containing 10 mM EDTA) at 37°C for 15 min. Crypts were isolated by shaking the fragments in pre-cold PBS buffer. The crypts were further digested in DMEM medium (Sigma-Aldrich) containing 1 mg/ml Collagenase t VIII (MCE # HY-E70005H) and 10 U/ml DNase (MCE # HY-108882) for 10 min at 37°C in a water bath. The resulting cell suspensions were filtered through a 75 μm cell strainer (Corning) and incubated for 1 h in ice with fixable cell live/death dye (MCE) to exclude dead cells. The live epithelial cells sorted from this process were used for subsequent experiments.

### Colonocyte apoptotic cell model

Colonocytes were cultured in completed DMEM medium (10% FBS, 100 U/ml PS) overnight, followed by the addition of TGF-β (30 ng/ml) to the medium for 24 h to establish an apoptotic cell model. Subsequently, the culture medium was replaced with fresh completed DMEM medium containing PBS (as control), 50 μM, 75 μM, 100 μM Resveratrol for 24 h. The cells were then used for apoptosis experiments and protein extraction.

### Detection of DNA fragmentation

Cells were lysed in lysis buffer and incubated for 30 min under room temperature. The lysate was then centrifuged at 300 *g* for 5 min, and the supernatant was added to streptavidin-coated microtiter plate. The plate was incubated at room temperature for at least 2 h. After incubation, the plates were washed by PBST 3 times. The amount of DNA fragments was quantified by measuring the peroxidase activity retained in the immunocomplex using 3-ethylbenzthiazolin-sulfonate substrate. The reaction was detected at a wavelength of 405 nm, normalized to a reference wavelength of 492 nm.

### Detection of the apoptotic cell by flow cytometry

Colonocytes were stained with FITC-conjugated annexin V and propidium iodide (Thermofisher, USA) and analyzed using FACS assays on an LSRFortessa instrument (BD Biosciences). Subsequent analysis of the results were performed on FlowJo software.

### Mitochondrial membrane potential

The mitochondrial membrane potential in colonocytes was assessed by the TMRE membrane potential assay (Catalog #701310, Cayman Chemical) with a working concentration of 50 nM. Fluorescence was measured using a Varioskan LUX microplate reader (Thermofisher, USA) with excitation and emission wavelengths set at 530 nm and 580 nm, respectively.

### Histology and immunofluorescence study

The collected colon was fixed in a 4% buffered paraformaldehyde solution (dissolved in water, v/v). After fixation, the tissues were dehydrated, embedded in paraffin, sectioned into 5 μm slices, and stained with Hematoxylin and Eosin. These stained sections were then examined under a light microscope and analyzed using ImageJ software. For the immunofluorescence experiment, antigen retrieval was performed by boiling the sections in sodium citrate buffer for 20 min. The sections were then blocked with 10% FBS in PBS for 1 hour at room temperature. Following, the sections were incubated at 4°C overnight with mouse anti-Occludin (ProteinTech, Cat# 27260-1-AP, 1/200 dilution) and anti-ZO-1 (ProteinTech, Cat# 21773-1-AP, 1/200 dilution) antibodies. The next day, fluorescent dye-conjugated secondary antibodies were applied for 1 h at room temperature. Finally, the sections were mounted with DAPI-Mountant (Thermofisher, USA). Fluorescence distribution in the colon was observed using a laser confocal microscope (Leica TCS MP8 CARS, Germany).

### RNA isolation

Total RNA was extracted by TRIzol reagent (Tiangen, China). Reverse transcription of 1 μg of RNA was performed by using the Reverse Transcription Mix (Thermofisher, USA). QPCR analyses were conducted on an Applied Biosystems PCR System, the specific primers listed in Supplementary Table S1.

### Protein analysis by immunoblotting

Colonocytes were lysed in RIPA buffer (BitoTech, China) supplemented with a protease inhibitor cocktail (MSE, USA). The lysates were subjected to SDS-PAGE, and proteins were transferred to a PVDF membrane (Catalog# 1620177, BIO-RAD) at 250 mA for 100 min. The membrane was blocked with 10% non-fat milk for at least 1 h, followed by incubation with the respective primary antibody at 4°C overnight. After washing with TBST 3 times, the membrane was incubated with respective secondary antibody for 2 h. The membrane was washed again with TBST for 3 times, and protein bands were detected using ECL reagents (Thermofisher, USA). Band intensity was quantified using ImageJ software. The antibodies used included anti-MDM2 (catalog # 27883-1-AP, ProteinTech), anti-P53 (catalog # 60283-2-Ig, ProteinTech), anti-PUMA (catalog # 55120-1-AP, ProteinTech), anti-BAX (catalog # 50599-2-Ig, ProteinTech), anti-BCL2 (catalog # 68103-1-Ig, ProteinTech), and anti-BETA-ACTIN (catalog # 81115-1-RR, ProteinTech).

### Statistical significance

All statistical analyses were performed using GraphPad Prism. Data are presented as mean ± SEM. For comparisons between groups, one-way ANOVA followed by Tukey’s *post hoc* test was used. To account for multiple comparisons, statistical significance was assessed using the Bonferroni correction, with a corrected p-value threshold of <0.05 considered statistically significant.

## Results

### Resveratrol alleviated the colonic damage caused by DSS-induced ulcerative colitis

**FIGURE 1:**
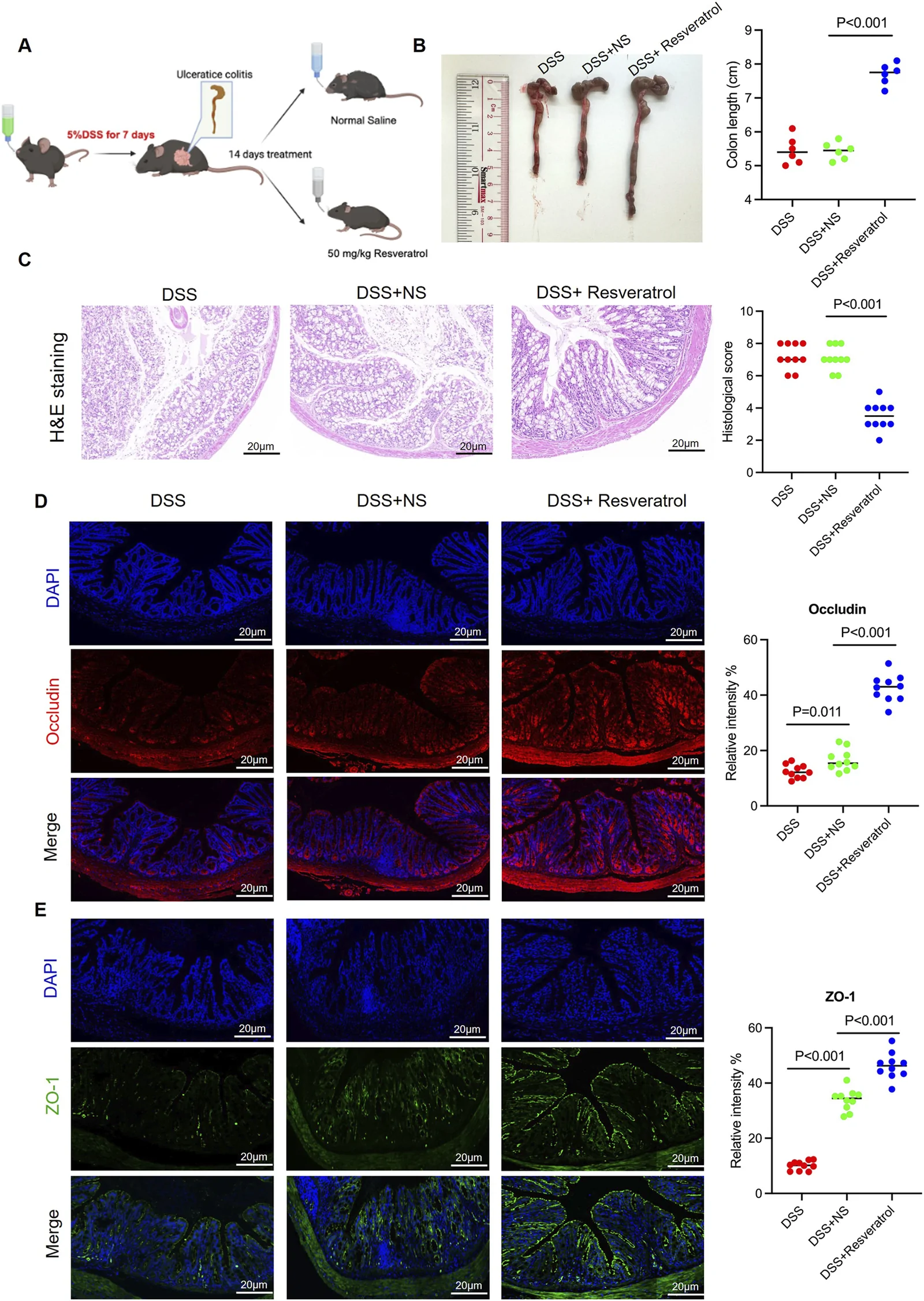
Resveratrol alleviated the colonic damage caused by DSS-induced ulcerative colitis. Eighteen C57B/L mice (male, 25 ± 2 g) were randomly divided into three groups (DSS, DSS + NS, DSS + Resveratrol). From day 1 to day 7, 5% (w/v) dextran sulfate sodium salt (DSS) was added to the drinking water of all groups. Mice in the DSS group were sacrificed on day 8. The remaining two groups were treated with normal saline (NS) and 50 mg/kg (w/v) Resveratrol from day 8 to day 24, respectively. On day 25, the remaining mice were sacrificed. **(A)** The experimental flow chart. **(B)** The colon length was measured and performed quantitively analysis (n = 6). **(C)** The colonic tissue of 3 groups were used to H&E staining and the histological score was judged by the person who did not familiar with this study (n = 10). **(D,E)** The colonic tissue was used to perform immunofluorescence staining by using antiZO-1 and anti-OCCLUDIN antibody, respectively. The relative intensity was analyzed by ImageJ (n = 10). The data are presented as mean ± SEM. Significance levels are denoted as *p < 0.05, **p < 0.01, and ***p < 0.001, indicating statistical significance.

Eighteen C57BL/6 mice were randomly divided into three groups: DSS, DSS+NS, and DSS+Resveratrol (n = 6 per group). The experimental procedure is shown in Figure 1A. After euthanizing the mice, the colons were collected for analysis. The results showed that colon length was significantly shortened in the DSS group, and 14 days of normal saline (NS) recovery did not restore colon length. However, after 14 days of Resveratrol treatment, colon length increased significantly compared to the NS group (P < 0.001) (Figure 1B). Hematoxylin and eosin (HE) staining revealed notable inflammatory cell infiltration in the colon sections of the DSS and DSS+NS groups, while Resveratrol treatment significantly improved this condition (Figure 1C). The permeability and integrity of the colon epithelial barrier were assessed by the expression of tight junction proteins. After 7 days of DSS-induced colitis, the expression of occludin and ZO-1 in the colon was markedly reduced. After 14 days of NS recovery, occludin expression did not significantly increase, but ZO-1 expression improved significantly (P < 0.001). In contrast, in the Resveratrol treatment group, both occludin and ZO-1 showed a marked increase (P < 0.001) (Figures 1D, E). These results indicate that 7 days of 5% DSS treatment successfully induces colitis in mice, and recovery with NS for 14 days does not improve colon damage. However, Resveratrol treatment significantly alleviates DSS-induced colitis, reduces inflammatory cell infiltration in colon tissue, increases tight junction protein expression, and thus enhances the integrity of the colon epithelium.

### Unrevealed the effects of Resveratrol on the colonic microenvironment based on single-cell sequencing

**FIGURE 2:**
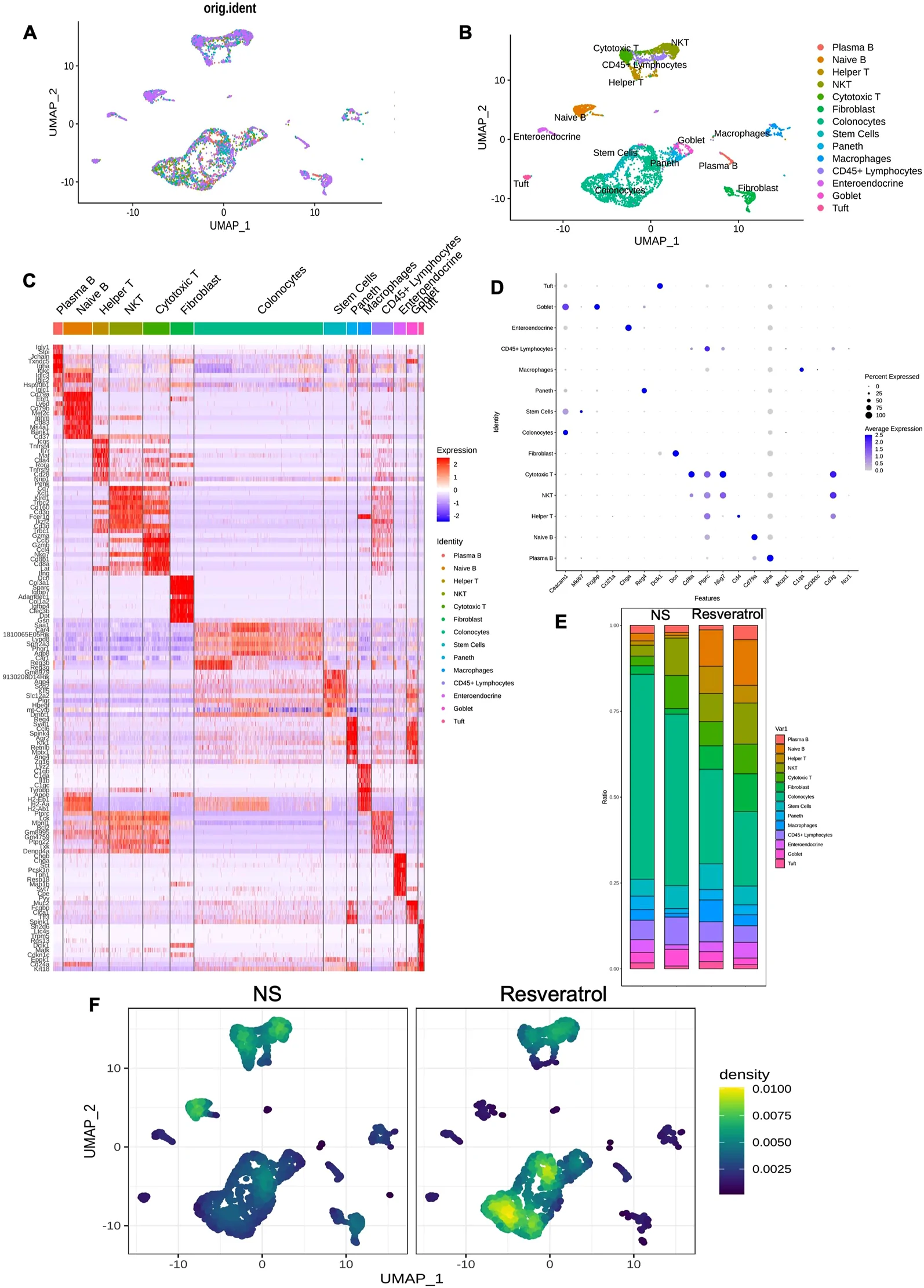
Unrevealed the effects of Resveratrol on the colonic microenvironment based on single-cell sequencing. The colonic tissue from NS and Resveratrol groups were used to perform single cell sequencing analysis. **(A)** Workflow for single-cell RNA sequencing of colonic tissues. **(B)** t-SNE plot showing 14 distinct cell populations. **(C-D)** Cell type annotation based on marker gene expression. **(E)** Proportional distribution of cell populations. **(F)** Heatmap showing changes in colonocyte intensity. The data are presented as mean ± SEM. Significance levels are denoted as *p < 0.05, **p < 0.01, and ***p < 0.001, indicating statistical significance.

To better understand the changes in the cellular environment of colonic tissue, we performed single-cell analysis on colonic tissues of NS and Resveratrol group mice. We generated scRNA-seq profiles of all cells isolated from the colonic tissues of normal saline (NS) and Resveratrol treatment groups after DSS-induced UC mice (Figure 2A). The scRNA-seq profiles were partitioned into 14 broad lineages, each identified by specific marker genes: Plasma B cells (Cd79a, Igha), Naive B cells (Cd79a, Ptprc), Helper T cells (Cd4, Cd3g), NK cells (Nkg7, Ncr1), Cytotoxic T cells (Cd8a, Cd3g), Fibroblasts (Dcn, Ccl21a), Colonocytes (Ceacam1, Fcgbp), Stem cells (Mki67), Paneth cells (Lyz1, Defa1), Macrophages (C1qa, Cd300c), CD45^+^ Lymphocytes (Ptprc, Cd3g), Enteroendocrine cells (Chga, Reg4), Goblet cells (Fcgbp, Muc2), and Tuft cells (Dclk1, Trpm5). These markers were used to annotate and distinguish the cell populations in the single-cell RNA sequencing analysis (Figure 2B–D). Subsequently, we performed the cell population between NC and Resveratrol group, it’s obvious that the colonocyte in NS group was decreased significantly (Figure 2E). The heatmap of cellular intensity also suggested the colonocytes intensity significantly changed between two groups (Figure 2E).

### Resveratrol inhibited the colonocyte apoptosis signal pathway in DSS-induced ulcerative colitis

**FIGURE 3:**
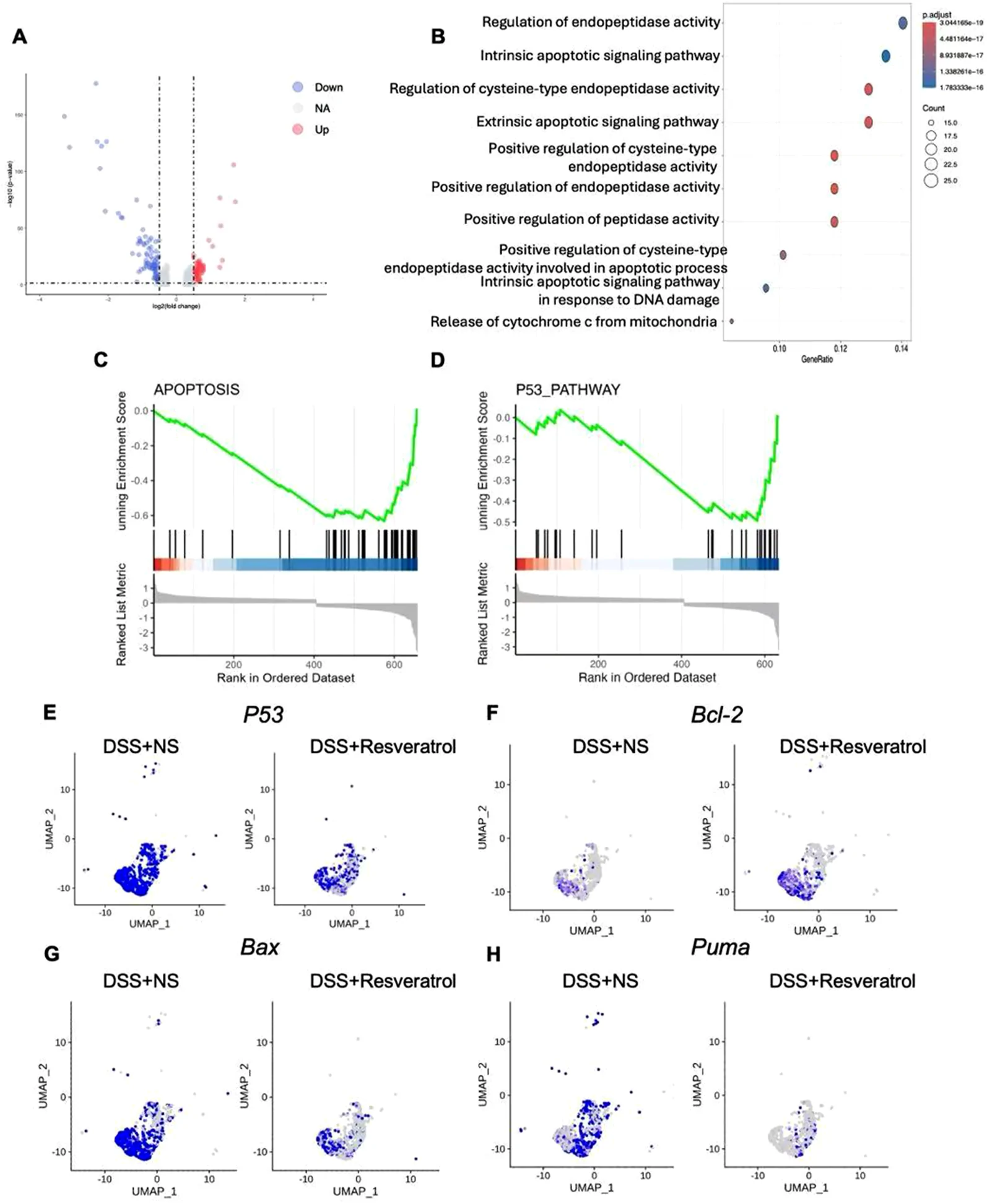
Resveratrol inhibited the colonocyte apoptosis signal pathway in DSS-induced ulcerative colitis. **(A)** The differential expressed genes (DEGs) were showed in the volcano map, upregulated with red color, downregulated with blue color. **(B)** the DEGs were used to perform KEGG enrichment analysis, the figure showed the top 10 pathway ways. **(C and D)** GSEA analysis were used to show the tendency involved in apoptosis pathway and P53 pathway. **(E–H)** the expression of P53, BCL2. BAX and PUMA in the colonocyte from two groups were showed. The data are presented as mean ± SEM. Significance levels are denoted as *p < 0.05, **p < 0.01, and ***p < 0.001, indicating statistical significance.

Because the above results show that Resveratrol has the greatest impact on the colonocytes at the cellular level in DSS-induced colitis mice compared to the NS group. Therefore, we focused on the differential expressed genes (DEGs) in colonocytes from two groups, the volcano map showed DEGs (p-value <1e−20 and |log2 (FC)| > 2) between 2 groups, upregulated (red) and downregulated (blue) DEGs in the Resveratrol groups are showed in the Figure 3A. Then we used these DEGs to perform KEGG enrichment analysis. The results showed most DEGs enriched on regulation of endopeptidase, intrinsic apoptotic signaling pathway, regulation of cysteine-type endopeptidase activity, extrinsic apoptotic signaling pathway, positive regulation of endopeptidase activity, signaling pathway in response to DNA damage and release of cytochrome c from mitochondria (Figure 3B). Gene Set Enrichment Analysis indicated that Resveratrol treatment induces the change of genes enriched on apoptosis and P53 pathway (Figures 3C, D). To verify the change of apoptosis between two groups, we examined the genes expression in the colonocytes between two groups. The results showed that P53 and pro-apoptotic gene Bax and Puma were significantly decreased in Resveratrol group, but anti-apoptotic gene Bcl2 was significantly increased in Resveratrol group (Figures 3E–H).

### Resveratrol suppressed the colonocyte apoptosis in a dose-dependent manner

**FIGURE 4:**
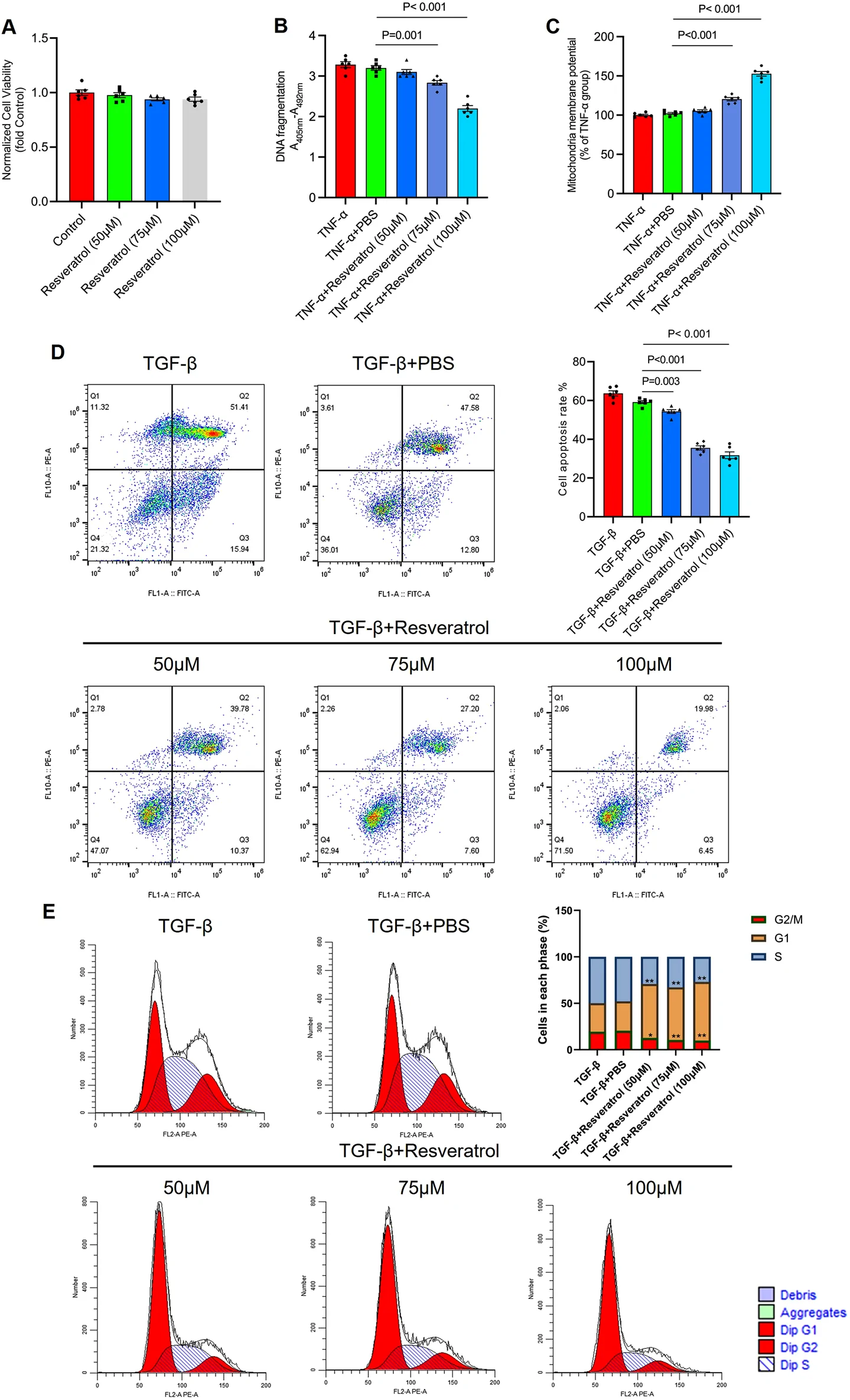
Resveratrol suppressed the colonocyte apoptosis in a dose-dependent manner. **(A)** Colonocytes isolated from health mice were treated with PBS (Control), 50, 75, and 100 nM Resveratrol and assessed cell viability using the MTT assay. **(B)** Colonocytes treated with TGF-β for 24 h to establish an apoptosis cell model, followed by treated with PBS (as Control), 50, 75, and 100 nm Resveratrol for 24 h respectively. **(B)** the DNA fragments was quantified by measuring the peroxidase activity retained in the immunocomplex using 3-ethylbenzthiazolin-sulfonate substrate. **(C)** the mitochondrial membrane potential was measured by TMRE assay. **(D)** Colonocytes were stained with FITC-conjugated annexin V and propidium iodide and analyzed by the flow cytometry to identify the apoptotic status. **(E)** Colonocytes were analyzed the cell cycle distribution using flow cytometry. The data are presented as mean ± SEM. Significance levels are denoted as *p < 0.05, **p < 0.01, and ***p < 0.001, indicating statistical significance.

To study the effect of Resveratrol on colonocyte apoptosis, we first treated colonocytes isolated from healthy mouse colon tissue with PBS (Control), 50, 75, and 100 nM Resveratrol and assessed cell viability using the MTT assay. The results showed that these concentrations of Resveratrol had no significant impact on colonocyte viability, confirming their suitability for subsequent experiments (Figure 4A). Next, we induced apoptosis in colonocytes by treating them with TGF-β for 24 h, followed by treatment with PBS, 50, 75, and 100 nM Resveratrol for 24 h. DNA fragmentation and mitochondrial membrane potential were assessed as indicators of apoptosis. Results showed that compared to the TGF-β group, DNA fragmentation and mitochondrial membrane potential did not change significantly in the PBS group. Among the Resveratrol-treated groups, 50 nM showed a trend toward improving DNA fragmentation and mitochondrial potential, though not significantly. However, 75 nM and 100 nM Resveratrol significantly reduced DNA fragmentation and increased mitochondrial membrane potential in colonocytes (FigureS 4B, C), with more pronounced effects at higher concentrations (P < 0.001). Flow cytometry analysis of annexin V/PI staining revealed significant apoptosis in the TGF-β (∼65%) and PBS (∼60%) groups, but Resveratrol treatment reduced apoptosis in a dose-dependent manner (Figure 4D). To further investigate the effects of Resveratrol on colonocytes, we analysed the cell cycle distribution using flow cytometry. The results revealed that Resveratrol treatment led to a significant increase in the proportion of cells in the G1 phase in a dose-dependent manner compared to the TGF-β and PBS control groups (Figure 4E). These results demonstrate that Resveratrol exerts a dose-dependent protective effect on colonocytes by reducing DNA fragmentation, enhancing mitochondrial membrane potential, and inhibiting excessive apoptosis. Additionally, Resveratrol induces cell cycle arrest at the G1 phase, which may provide cells with additional time to repair damage and prevent the initiation of apoptotic pathways. Together, these findings suggest that Resveratrol not only mitigates apoptosis but also promotes cellular repair and survival. Based on these observations, the 100 nM concentration of Resveratrol, which showed the most pronounced effects, was selected for subsequent experiments to further investigate its mechanisms of action.

### Resveratrol targeting MDM2/P53/PUMA axis to inhibit the apoptosis

**FIGURE 5:**
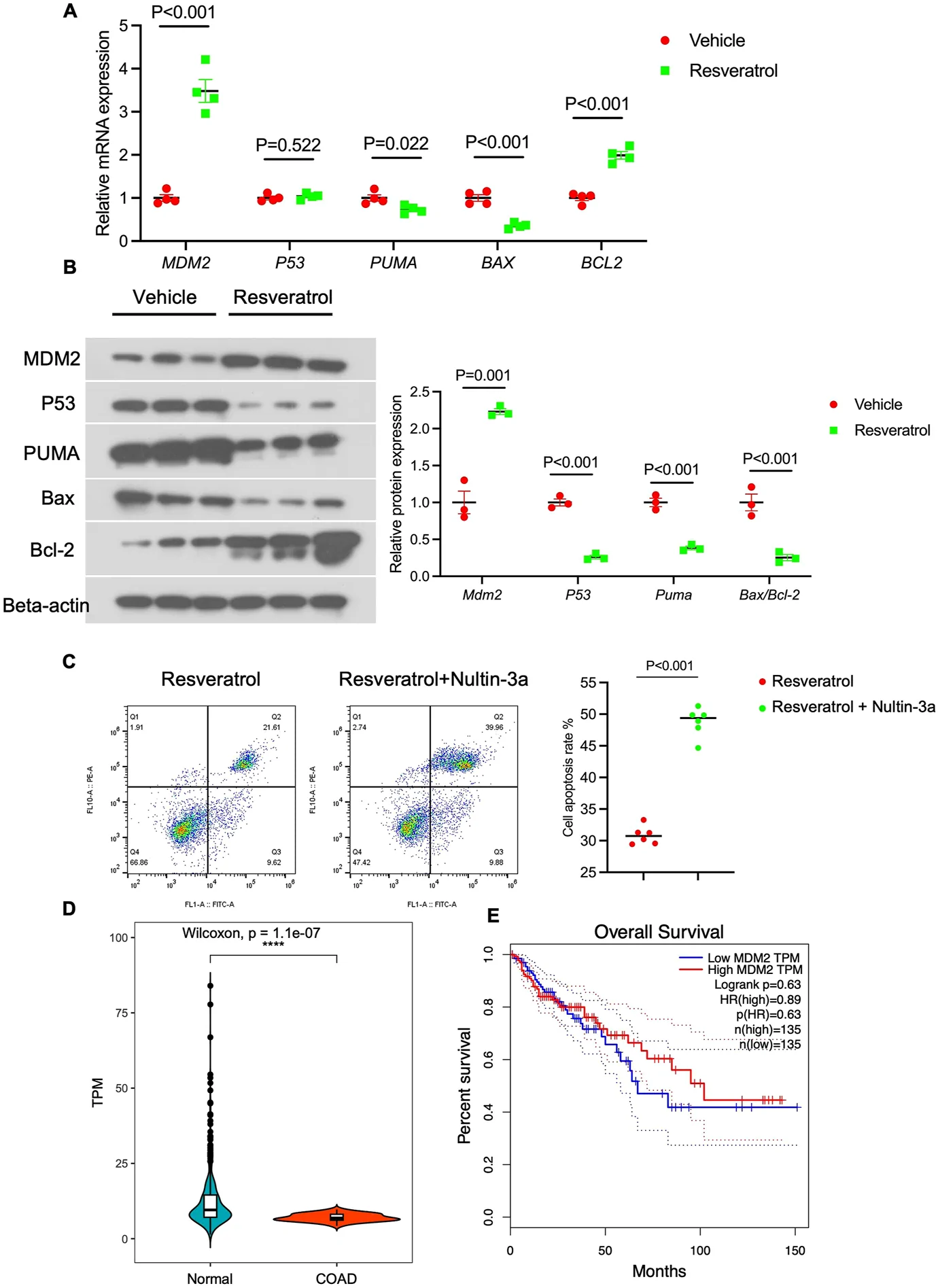
Resveratrol targeting MDM2/P53/PUMA axis to inhibit the apoptosis. Colonocytes isolated from health mice were treated with TGF-β for 24 h to establish an apoptosis cell model, followed by treated with PBS (as Control) and 100 nm Resveratrol for 24 h respectively. **(A)** the mRNA expression of MDM2, P53, PUMA, BAX and BCL2 were detected by QPCR analysis (n = 4). **(B)** The protein expression of MDM2, P53, PUMA, BAX and BCL2 were detected by immunoblotting experiment, normalized by the expression of BETA-ACTIN, n = 4. **(C)** Colonocytes were treated with TGF-β for 24 h, followed by treated with 100 nm Resveratrol with/without Nultin-3a for 24 h, the Colonocytes were stained with FITC-conjugated annexin V and propidium iodide and analyzed by the flow cytometry to identify the apoptotic status. **(D)** the expression of MDM2 in normal tissue and colon adenocarcinoma (COAD) from TCGA database. **(E)** the overall survival predication of high MDM2 expression and low MDM2 expression. The data are presented as mean ± SEM. Significance levels are denoted as *p < 0.05, **p < 0.01, and ***p < 0.001, indicating statistical significance.

Single-cell data indicated that Resveratrol altered the P53 signaling pathway in colonocytes. We assessed the expression of MDM2 (P53 regulator), P53, and downstream targets (PUMA, BAX, BCL2) at both mRNA and protein levels. qPCR results showed that Resveratrol significantly increased MDM2 expression and reduced P53 and its downstream genes (Figure 5A), consistent with immunoblotting results (Figure 5B). To confirm whether the MDM2/P53/PUMA axis is key in Resveratrol’s anti-apoptotic effect, we treated colonocytes with Resveratrol and Resveratrol + Nutlin-3a (an MDM2 inhibitor) after TNF-α-induced apoptosis. The results showed that Nutlin-3a abolished Resveratrol’s inhibitory effect on apoptosis (Figure 5C), indicating MDM2 is critical in Resveratrol-mediated apoptosis regulation. TCGA analysis revealed that MDM2 expression was significantly reduced in colon cancer patients (Figure 5D), and high MDM2 expression correlated with better survival outcomes (Figure 5E). These findings suggest that Resveratrol promotes P53 degradation by increasing MDM2, downregulating the apoptosis pathway, and identifying MDM2 as a potential target for colon cancer treatment.

### Overexpressed MDM2 by Adeno-associated viruses can protect the colonic damage from DSS treatment

**FIGURE 6:**
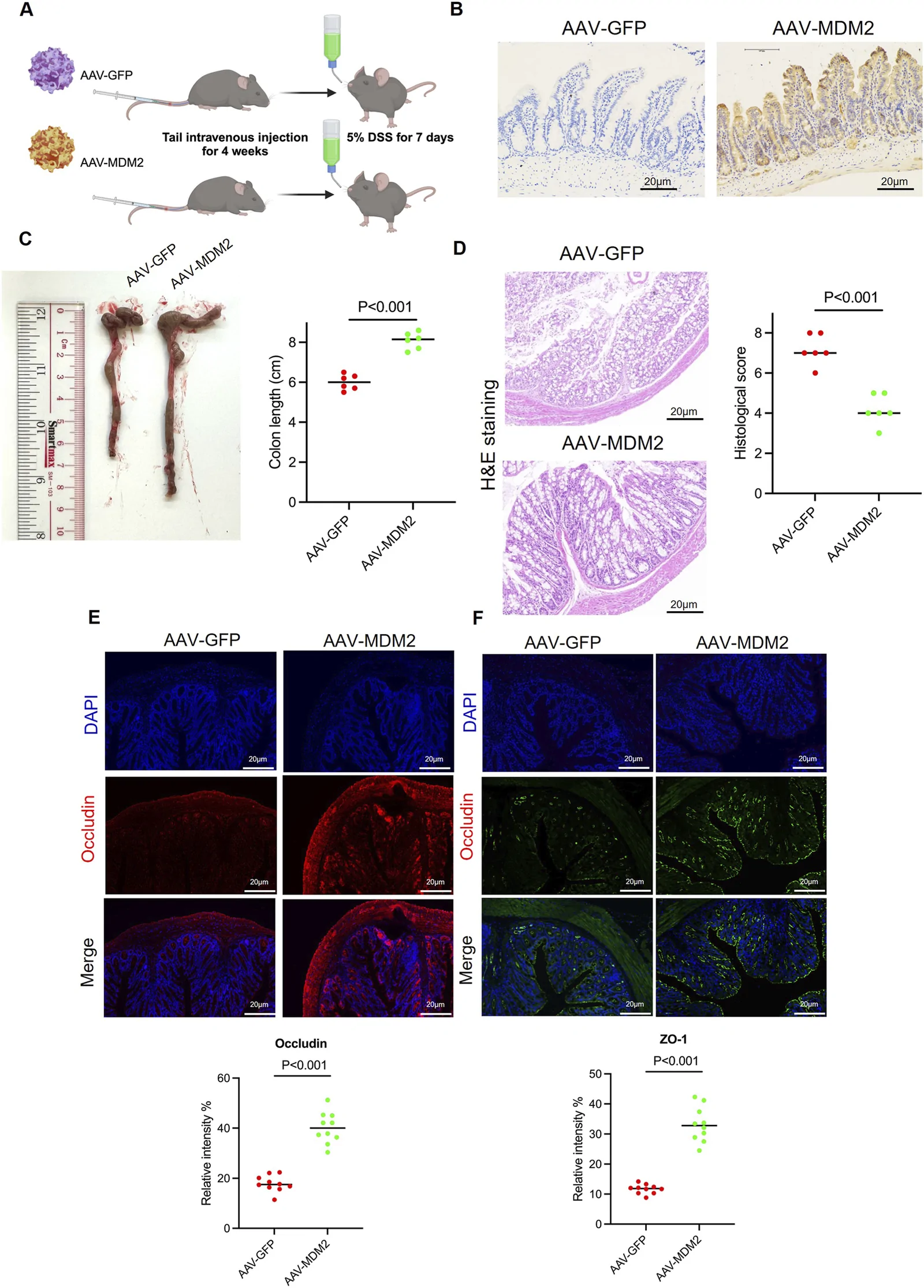
Overexpressed MDM2 by Adeno-associated viruses can protect the colonic damage from DSS treatment. Twelve C57B/L mice (male, 25 ± 2 g) were randomly divided into two groups (AAV-GFP and AAV-MDM2). Mice in both groups were infected with Adeno-Associated Virus (AAV) expressing either GFP or MDM2 for 4 weeks. Subsequently, 5% (w/v) DSS was added to the drinking water for 7 days, and the mice were then sacrificed. **(A)** The experimental flow chart. **(B)** The IHC staining by using anti-MDM2 antibody in colon tissue. **(C)** The colon length was measured and performed quantitively analysis (n = 6). **(D)** The colonic tissue of 2 groups were used to H&E staining and the histological score was judged by the person who did not familiar with this study (n = 6). **(E,F)** The colonic tissue was used to perform immunofluorescence staining by using anti-ZO-1 and anti-OCCLUDIN antibody, respectively. The relative intensity was analyzed by ImageJ (n = 10). The data are presented as mean ± SEM. Significance levels are denoted as *p < 0.05, **p < 0.01, and ***p < 0.001, indicating statistical significance.

The above results confirm that Resveratrol inhibits P53-mediated pro-apoptotic signaling by increasing MDM2 expression. TCGA analysis also showed higher MDM2 expression in healthy colon tissues, prompting us to investigate whether MDM2 overexpression could protect mice from DSS-induced colonic damage. We constructed AAV-MDM2 and AAV-GFP (control) vectors, infected mice via tail vein injection for 4 weeks, and then treated with 5% DSS for 7 days (Figure 6A). IHC staining confirmed that AAV-MDM2 significantly increased MDM2 expression in colon tissues (Figure 6B). Dissection showed that the colon length was longer in the AAV-MDM2 group compared to the AAV-GFP group (Figure 6C). HE staining revealed that MDM2 overexpression alleviated DSS-induced damage, reduced mucosal thinning, and decreased inflammatory cell infiltration (Figure 6D). Immunofluorescence analysis showed significant increases in tight junction proteins (occludin and ZO-1) in the AAV-MDM2 group (P < 0.001) (Figures 6E, F), indicating improved colon integrity. These results suggest that MDM2 overexpression protects against DSS-induced colonic damage, positioning MDM2 as a potential therapeutic target for ulcerative colitis.

## Discussion

In UC, pro-inflammatory cytokines such as tumor necrosis factor-alpha (TNF-α), interleukin-1 beta (IL-1β), and interferon-gamma (IFN-γ) are elevated (Tatiya-Aphiradee et al., 2018). These cytokines can induce apoptosis in colonocytes by activating death receptors and downstream signaling pathways, such as the caspase cascade (Man et al., 2017). Chronic inflammation in UC leads to the production of reactive oxygen species (ROS), which can cause oxidative damage to cellular components, including DNA, proteins, and lipids (Guo et al., 2022). This oxidative stress can also trigger apoptosis in colonocytes too (Luo et al., 2019). Besides, In UC, mitochondrial dysfunction, characterized by the loss of mitochondrial membrane potential and the release of cytochrome c, can activate caspases and promote colonocyte apoptosis (Verma et al., 2022). Therefore, the interplay between ulcerative colitis, intestinal barrier dysfunction, and colonocyte apoptosis is a key area of research.

Our findings align with and extend previous studies on the protective effects of Resveratrol in inflammatory bowel disease (IBD) and colitis models. Resveratrol has been widely reported to exhibit anti-inflammatory, antioxidant, and anti-apoptotic properties, which are consistent with our observations. For instance, studies have shown that Resveratrol reduces colonic inflammation and oxidative stress in DSS-induced colitis by modulating NF-κB and Nrf2 signaling pathways (Ma et al., 2023; Alavi et al., 2021). Similarly, our results demonstrate that Resveratrol significantly attenuates DSS-induced colonic damage and apoptosis, further supporting its role as a potent therapeutic agent in colitis.

However, our study provides novel insights by identifying the MDM2/p53/PUMA axis as a key mechanism through which Resveratrol exerts its anti-apoptotic effects. While previous studies have primarily focused on Resveratrol’s ability to inhibit NF-κB or activate Nrf2, our single-cell RNA sequencing data reveal that Resveratrol specifically targets the p53 signaling pathway to regulate apoptosis. This finding is consistent with reports that p53 activation plays a critical role in stress-induced apoptosis (Gottlieb and Oren, 1998; Yu et al., 2019), but our work uniquely highlights the involvement of MDM2 and PUMA in mediating Resveratrol’s protective effects. This adds a new dimension to the understanding of Resveratrol’s mechanisms of action in colitis.

In contrast to some studies that suggest p53 activation promotes apoptosis in response to cellular stress (Lee et al., 2020), our results show that Resveratrol inhibits excessive apoptosis by modulating the MDM2/p53/PUMA axis. This discrepancy may be due to differences in experimental models or the timing of Resveratrol administration. For example, in cancer models, p53 activation often leads to apoptosis, whereas in colitis, the suppression of p53-mediated apoptosis by Resveratrol appears to protect epithelial cells. This dual role of p53 underscores its context-dependent functions and highlights the importance of targeting specific pathways in different disease settings.

Additionally, our observation that MDM2 overexpression protects against DSS-induced damage is supported by studies showing that MDM2 plays a crucial role in regulating p53 activity and cell survival (Haupt et al., 1997; Zafar et al., 2021). However, our findings diverge from some reports suggesting that MDM2 overexpression may promote tumorigenesis by inhibiting p53 (Nayak et al., 2018). This difference may reflect the distinct roles of MDM2 in acute inflammation versus cancer. In the context of colitis, MDM2 appears to exert a protective effect by modulating p53-mediated apoptosis, whereas in cancer, its overexpression may contribute to p53 inactivation and tumor progression. This highlights the need for context-specific therapeutic strategies targeting MDM2.

Finally, our clinical database analysis showing that high MDM2 expression correlates with better survival outcomes in colon cancer patients aligns with some studies suggesting that MDM2 may have p53-independent roles in promoting cell survival (Bhatia et al., 2023). However, this contrasts with other reports indicating that MDM2 overexpression is associated with poor prognosis in certain cancers (Ding et al., 2019; Ye et al., 2021). These differences may stem from variations in cancer types, stages, or patient populations, emphasizing the complexity of MDM2’s role in disease progression.

## Conclusion and recommendations

Our study demonstrates that resveratrol protects against DSS-induced colitis by inhibiting excessive colonocyte apoptosis through the MDM2/p53/PUMA axis, preserving intestinal barrier integrity and reducing colonic damage. These findings highlight Resveratrol’s therapeutic potential for ulcerative colitis (UC) and position MDM2 as a promising target, supported by clinical data showing that high MDM2 expression correlates with better survival outcomes in colon cancer. Moving forward, we recommend further mechanistic studies in chronic colitis models, exploration of Resveratrol’s clinical translation, and investigation of MDM2-targeted therapies for both UC and colon cancer. Additionally, evaluating Resveratrol in combination with existing treatments and exploring the broader implications of MDM2 modulation could pave the way for innovative therapeutic strategies.

## Data Availability

The original contributions presented in the study are publicly available. This data can be found here: [https://www.ncbi.nlm.nih.gov/bioproject/PRJNA1254630].
