# Correction: Resveratrol targeting MDM2/P53/PUMA axis to inhibit colonocyte apoptosis in DSS-induced ulcerative colitis mice

**DOI:** 10.3389/fphar.2026.1983201

**Published:** 2026-09-25

**Authors:** Rui Tang, Ling Jiang, Quan Ji, Pengyuan Kang, Yuan Liu, Pengyu Miao, Xiaofan Xu, Mingxi Tang

**Affiliations:** 1 Department of Pathology, Yaan People’s Hospital, Yaan, China; 2 School of Basic Medical Sciences, Southwest Medical University, Luzhou, China; 3 Department of Anesthesiology Management, the Affiliated Hospital of Southwest Medical University, Luzhou, China; 4 Department of Microbiology and Infectious Disease Center, School of Basic Medical Sciences, Peking University, Beijing, China; 5 Department of Health Technology and Informatics, The Hong Kong Polytechnic University, Kowloon, China; 6 Department of Pathology, The Affiliated Hospital, Southwest Medical University, Luzhou, China; 7 Precision Medicine Center, Yaan People’s Hospital, Yaan, China

**Keywords:** ulcerative colitis, resveratrol, apoptosis, colonocyte, MDM2/P53/PUMA axis

There was a mistake in Figures 1, 6 as published. Representative images of stained sections were misused. The corrected Figures 1, 6 appear below.

The original version of this article has been updated.

**FIGURE 1:**
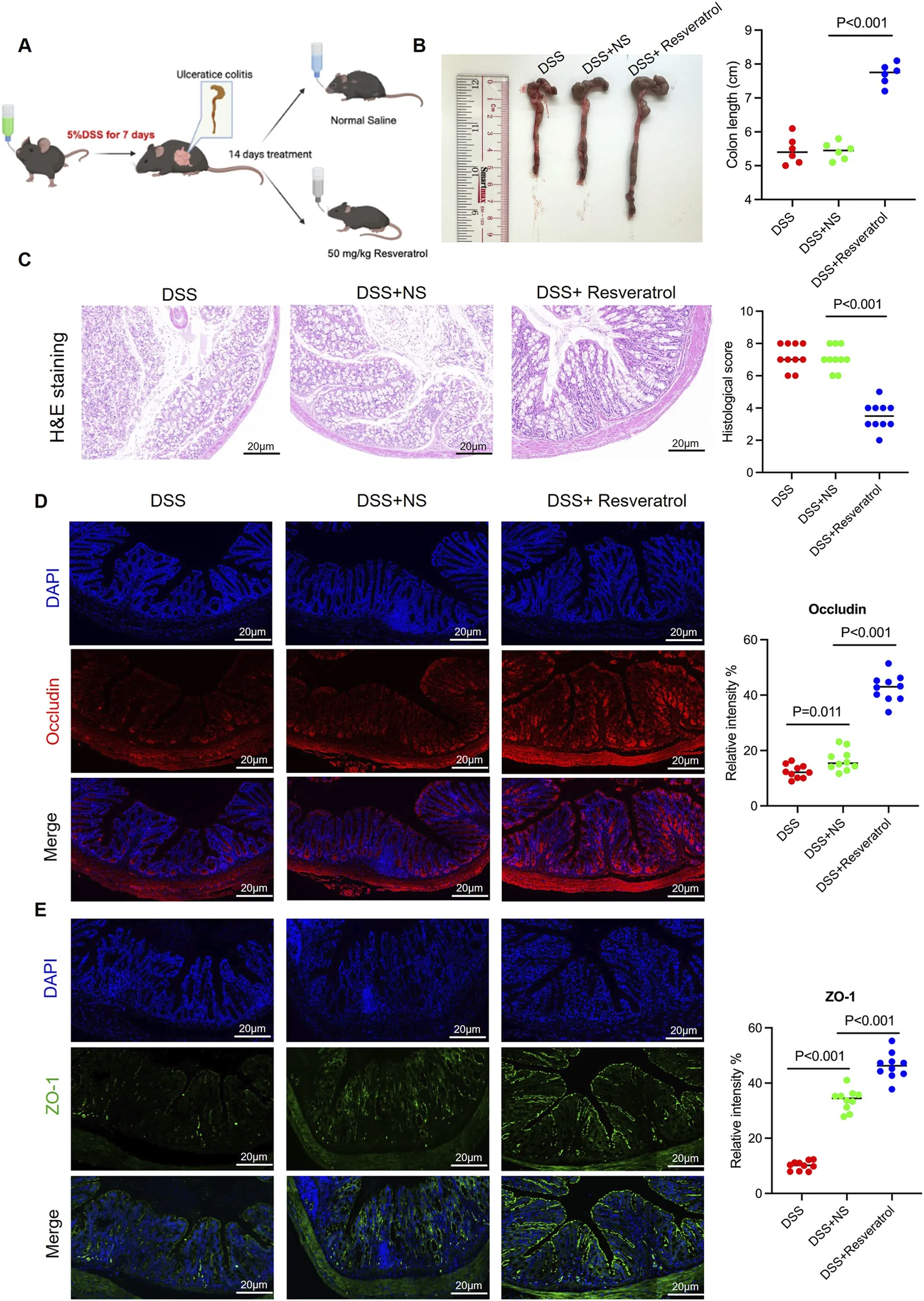
Resveratrol alleviated the colonic damage caused by DSS-induced ulcerative colitis. Eighteen C57B/L mice (male, 25 ± 2 g) were randomly divided into three groups (DSS, DSS + NS, DSS + Resveratrol). From day 1 to day 7, 5% (w/v) dextran sulfate sodium salt (DSS) was added to the drinking water of all groups. Mice in the DSS group were sacrificed on day 8. The remaining two groups were treated with normal saline (NS) and 50 mg/kg (w/v) Resveratrol from day 8 to day 24, respectively. On day 25, the remaining mice were sacrificed. **(A)** The experimental flow chart. **(B)** The colon length was measured and performed quantitively analysis (n = 6). **(C)** The colonic tissue of 3 groups were used to H&E staining and the histological score was judged by the person who did not familiar with this study (n = 10). **(D,E)** The colonic tissue was used to perform immunofluorescence staining by using antiZO-1 and anti-OCCLUDIN antibody, respectively. The relative intensity was analyzed by ImageJ (n = 10). The data are presented as mean ± SEM. Significance levels are denoted as *p < 0.05, **p < 0.01, and ***p < 0.001, indicating statistical significance.

**FIGURE 6:**
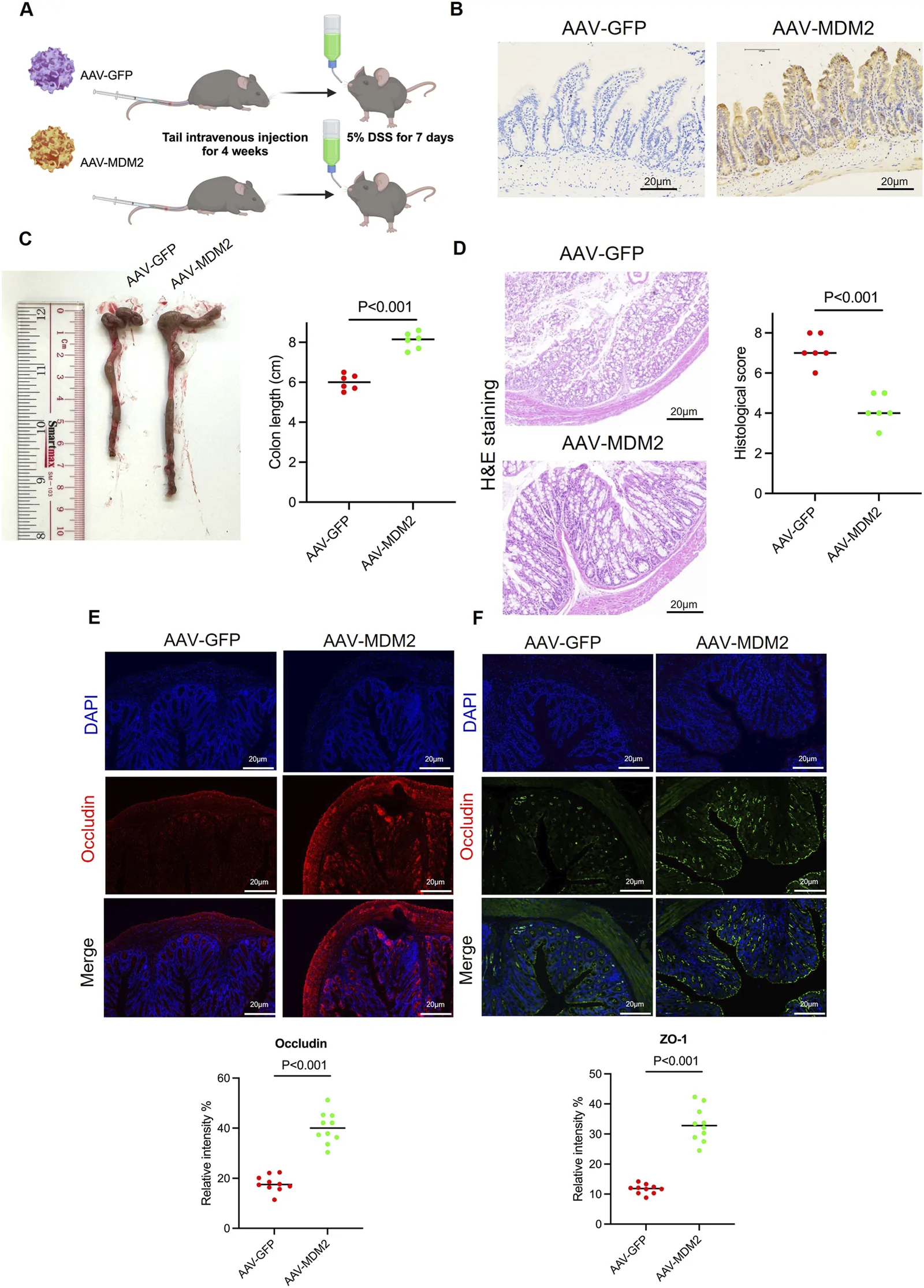
Overexpressed MDM2 by Adeno-associated viruses can protect the colonic damage from DSS treatment. Twelve C57B/L mice (male, 25 ± 2 g) were randomly divided into two groups (AAV-GFP and AAV-MDM2). Mice in both groups were infected with Adeno-Associated Virus (AAV) expressing either GFP or MDM2 for 4 weeks. Subsequently, 5% (w/v) DSS was added to the drinking water for 7 days, and the mice were then sacrificed. **(A)** The experimental flow chart. **(B)** The IHC staining by using anti-MDM2 antibody in colon tissue. **(C)** The colon length was measured and performed quantitively analysis (n = 6). **(D)** The colonic tissue of 2 groups were used to H&E staining and the histological score was judged by the person who did not familiar with this study (n = 6). **(E,F)** The colonic tissue was used to perform immunofluorescence staining by using anti-ZO-1 and anti-OCCLUDIN antibody, respectively. The relative intensity was analyzed by ImageJ (n = 10). The data are presented as mean ± SEM. Significance levels are denoted as *p < 0.05, **p < 0.01, and ***p < 0.001, indicating statistical significance.

